# Association between inflammatory bowel disease and Parkinson’s disease: a prospective cohort study of 468,556 UK biobank participants

**DOI:** 10.3389/fnagi.2023.1294879

**Published:** 2024-01-15

**Authors:** Hai-li Wang, Zhi-yun Wang, Jie Tian, Dong-rui Ma, Chang-he Shi

**Affiliations:** ^1^Department of Surgery ICU, The First Affiliated Hospital of Zhengzhou University, Zhengzhou University, Zhengzhou, Henan, China; ^2^Department of Neurology, The First Affiliated Hospital of Zhengzhou University, Zhengzhou University, Zhengzhou, Henan, China; ^3^Zhengzhou Railway Vocational and Technical College, Zhengzhou, Henan, China

**Keywords:** Parkinson’s disease, inflammatory bowel disease, prospective cohort study, propensity score matching, polygenic risk score

## Abstract

**Introduction:**

Inflammatory Bowel Disease (IBD) and Parkinson’s disease (PD) are both chronic, progressive disorders. As such, given the inconclusive results of extensive research on the association between IBD and PD, our study intends to examine this relationship further using the UK Biobank database.

**Methods:**

We conducted a prospective cohort study using the Cox proportional hazards model, analyzing data from the UK Biobank to investigate the relationship between IBD and PD, following subjects until PD diagnosis, loss to follow up, death or study termination on 30 June, 2023.

**Results:**

The results show that IBD had no effect on the risk of PD (HR: 1.356, 95% CI: 0.941–1.955, *p* = 0.103), and the effect remained consistent in specific Crohn’s disease, ulcerative colitis or unclassified IBD populations. In addition, after sensitivity analysis using propensity matching scores and excluding patients diagnosed with PD 5 or 10 years after baseline, IBD had no effect on the risk of PD. However, in the subgroup analysis, we found that in females (HR: 1.989, 95% CI: 1.032–3.835, *p* = 0.040), the polygenic risk score was highest (HR: 2.476, 95% CI: 1.401–4.374, *p* = 0.002), and having ulcerative colitis without hypertension (HR: 2.042, 95% CI: 1.128–3.697, *p* = 0.018) was associated with an increased risk of PD.

**Conclusion:**

In conclusion, over an average follow-up period of 13.93 years, we found no significant association between IBD and PD.

## 1 Introduction

Parkinson’s disease (PD) ranks as the second-most prevalent neurodegenerative disorder globally (Tolosa et al., 2021). With the anticipated influx of elder populations, both the prevalence and associated global healthcare burden of PD are expected to rise substantially (Collaborators, 2019). Classified as a multifactorial condition, PD emerged from the interaction of genetic predispositions, environmental factors, and immune mechanisms. In addition to being characterized by motor symptoms, including resting tremor, bradykinesia, muscular rigidity, and postural instability (Hawkes et al., 2007), a range of non-motor manifestations, including gastrointestinal dysfunction, anxiety, and insomnia, have been observed to precede the onset of the aforementioned motor symptoms in PD by several years (Gallagher et al., 2010). The hallmark features of PD include the progressive loss of dopaminergic neurons within the substantia nigra and the emergence of Lewy bodies, which are aggregates primarily composed of abnormally folded α-synuclein (α-syn) (Kalia and Lang, 2015). Intriguingly, Lewy bodies have been observed to manifest at early stages within the gastrointestinal tract of individuals diagnosed with PD, thereby lending credence to existing theories regarding gastrointestinal origins of the disease (Guarino et al., 2008; Lebouvier et al., 2010; Shannon et al., 2012). According to Braak’s pioneering hypothesis, the gastrointestinal tract is proposed as the initial site of PD pathogenesis, where an unidentified pathogen penetrates the gastric mucosal barrier, initiating the aggregation of α-syn. Subsequently, this aggregation is believed to propagate to the central nervous system via the vagus nerve in a manner akin to prions, traveling retrograde along neuronal axons, ascending from the lower brain stem through the medulla oblongata and midbrain, and eventually reaching the cerebral cortex (Braak et al., 2003). Significant changes in the gut microbiome (Rogers et al., 2016; Cryan et al., 2019; Morais et al., 2021), which is crucial to the gut-brain axis and observed in both PD and Inflammatory Bowel Disease (IBD) (Burokas et al., 2015; Weingarden and Vaughn, 2017; Lee et al., 2021; Wang et al., 2021), suggest a potential impact on the progression of these conditions, lending further support to this hypothesis.

IBD is characterized by chronic, recurrent inflammation of the intestines (Zhang, 2014), encompassing ulcerative colitis (UC) and Crohn’s disease (CD) (Harris and Chang, 2018; Younis et al., 2020). The etiology of IBD is considered multifactorial, likely stemming from the complex interplay among genetic predisposition, environmental influences, and altered gut microbiota (Torres et al., 2017), all of which contribute to dysregulation in both innate and adaptive immune responses, contributing to the current upward trajectory of IBD (Larsen et al., 2023). Individuals diagnosed with PD demonstrate an increase in pro-inflammatory factors within the gastrointestinal tract. Both IBD and PD exhibit similar alterations in gut microbiota, including changes in key bacterial communities and reduced butyrate levels, which in turn contribute to the impairment of intestinal mucosal integrity (Romano et al., 2021). Mutations in leucine-rich repeat kinase 2 (LRRK2), which are associated with PD (Myasnikov et al., 2021), have also recently been reported to connected with IBD (Lee et al., 2021). In addition to gastrointestinal symptoms, IBD can also cause neurological effects in both the central and peripheral nervous systems, potentially coinciding with such conditions as PD, Multiple Sclerosis, and stroke (Ferro and Oliveira, 2021). Furthermore, the observed correlation between IBD and PD, particularly in terms of genetic overlap, inflammatory processes, and intestinal permeability, may suggest a link between the two conditions (Devos et al., 2013; Li et al., 2021).

**Figure 1:**
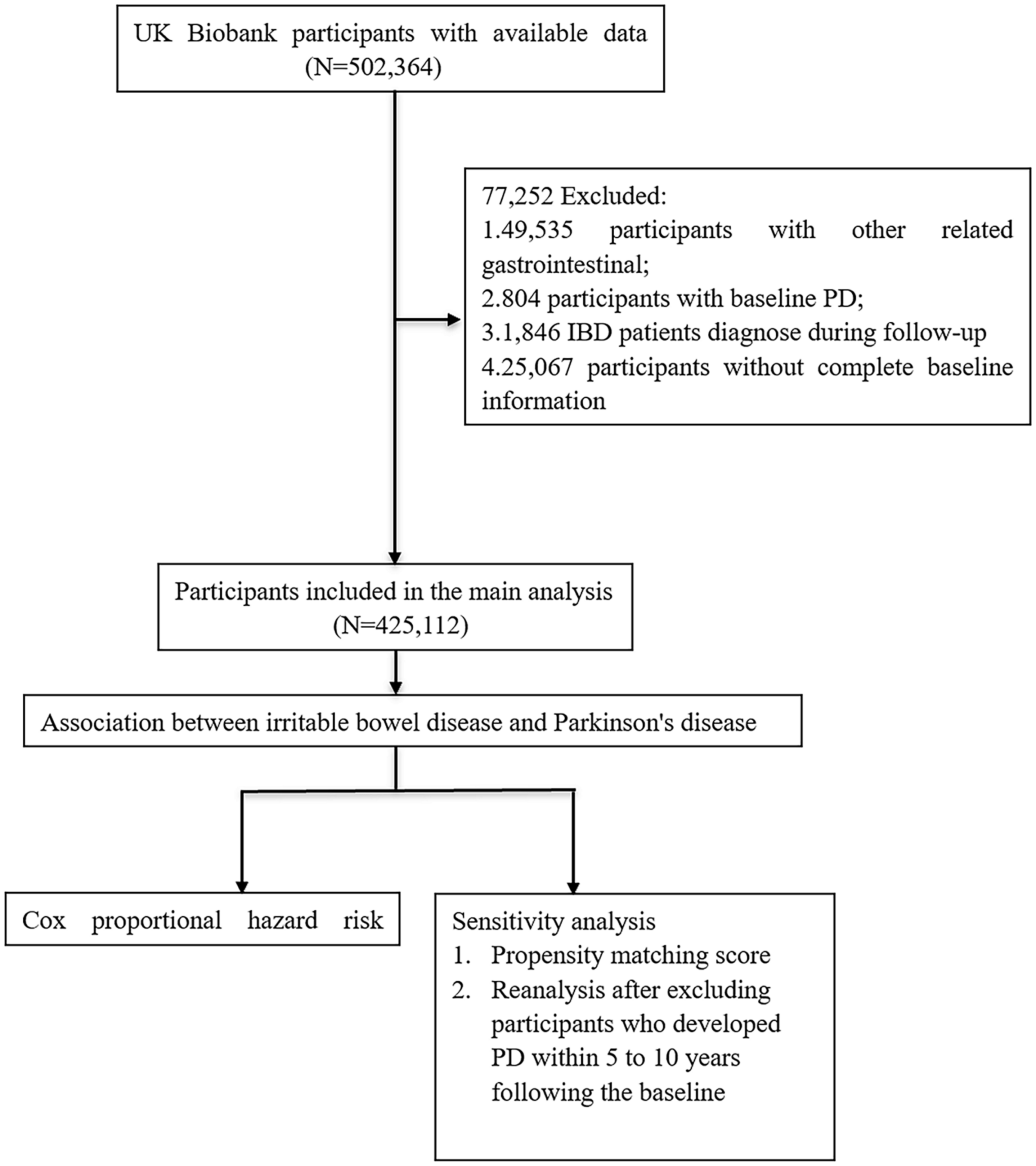
Analytical workflow for the research. PD, Parkinson’s disease; IBD, inflammatory bowel disease.

Utilizing participant data from the UK Biobank, we conducted a large-scale prospective cohort study employing Cox proportional hazard model to investigate the association between IBD and PD. In addition, propensity score matching (PSM) was implemented to adjust for confounding variables and to mitigate their interference in the analysis (Figure 1). Consequently, after excluding participants who developed PD within 5 or 10 years following the baseline, the data were subsequently reanalyzed.

## 2 Materials and methods

### 2.1 Study population

The UK Biobank is a large prospective cohort study that collected genetic, physical and health data from more than 500,000 participants aged 40 to 69 from 22 assessment centers across the UK between 2006 and 2010, with a response rate of 5.47 percent. The richness and comprehensiveness of the data enable investigators to conduct an extensive array of epidemiological research, and we can browse the data of UK Biobank through its website.^1^ In addition, the UK Biobank study was approved by the Northwest Multi-Center Research Ethics Committee, and all participants signed written informed consent.

Among all 502,364 participants in the UK Biobank study, we first excluded participants (*n* = 49,535) with other gastrointestinal diseases (Supplementary Table 1). Further, after calculating the time interval between PD or IBD diagnosis and inclusion in the study, we further excluded participants who had PD at the baseline (*n* = 804) and who were diagnosed with IBD during the follow-up period (*n* = 1,846), so all IBD patients were diagnosed before baseline and participants were diagnosed with PD during follow-up, establishing that all recorded instances of PD transpired subsequent to an IBD diagnosis. Finally, we excluded participants with incomplete baseline data (*n* = 25,067), leaving 425,112 participants in total to be included in the final study.

### 2.2 Definition of IBD and PD

IBD, encompassing CD (K50) and UC (K51), along with PD (G20), was identified based on the International Classification of Diseases, Tenth Revision (ICD-10) codes. The diagnostic records were sourced from primary care databases, self-reported medical histories and hospital admission records, and we divided the participants into a healthy control group and an IBD group. Thereafter, the IBD group was further categorized into three distinct subgroups based on specific diagnostic criteria: “CD”, “UC”, and “IBD-U”, where the latter refers to participants diagnosed with both CD and UC concurrently.

### 2.3 Covariates

In the baseline characteristics section, information on participants’ age (a continuous variable), gender (male/female), and Townsend Deprivation Index score (a continuous variable) is available. The Townsend Deprivation Index is calculated based on the most recent national census output areas, employing the postal codes available at the time of participant enrolment in the UK Biobank study. This provides a corresponding deprivation score for each respective geographic location, offering a measure for assessing the level of socioeconomic deprivation in a population, where higher index values indicate greater socioeconomic deprivation. The data collected via the Assessment Centre’s touchscreen interface also include such variables such as ethnicity (White/Non-White), education level (University/Non-University), smoking status (Never/Previous/Current), and frequency of alcohol intake (0/1/2/3/4), where scoring for the latter is determined as follows: ‘0’ denotes abstinence or drinking only on special occasions; “1” indicates 1–3 instances per month; “2” suggests 1–2 instances per week; “3” implies 3–4 instances per week; and “4” represents daily or near-daily consumption. Regarding education level, we define a “university” education as possessing a “college or university degree”, whereas all other categories are defined as “non-university”. In addition, covariates also include Body Mass Index (BMI, a continuous variable) as well as the presence of diabetes (coded as E10-E14) and hypertension (coded as I10-I15) based on the ICD-10 classifications.

In addition, the UK Biobank contains polygenic risk scores (PRSs) for 25 quantitative traits and 25 diseases, demonstrating a good and stable performance in predicting individual disease risk (Lewis and Vassos, 2020). The PRS serves as an effective tool for clinical risk prediction, functioning by evaluating individual variations in multiple genetic loci to predict the risk of specific diseases (Lu et al., 2022), where the higher the score, the greater the risk of developing related diseases. We used the Standard PRS for PD to assess an individua’s risk of developing PD at the genetic level, and risk categories were stratified among all 502,364 participants, the top 25% of whom were defined as low risk, 25–75% as moderate risk, and the bottom 25% as high risk. Ultimately, we included 11 covariates in total.

### 2.4 Statistical analysis

Continuous variables of the baseline characteristics are presented as means and standard deviations, and categorical variables are presented as percentages. For continuous variables, the analysis of variance was used, while for categorical variables, the chi-square test was used to compare the baseline characteristics of the two groups, and a baseline table was drawn.

Cox proportional hazards models were used to test the association between IBD and PD, with end point events including the first PD diagnosis, loss to follow-up, death or end of the study (June 2023), after which, the time interval from baseline to end point events was calculated. In our analysis, the hazard ratio (HR) value of the first level of the categorical variables was normalized to 1, serving as a reference for comparison with other levels. Then, we performed a survival analysis of the healthy control and IBD groups, drew survival curves using the Kaplan–Meier method and used the Log-rank test to examine the difference. Univariate Cox regression analysis was conducted to include variables that were statistically significant, which were then incorporated into a multivariate Cox regression model, and forest plots were drawn. Three multivariate Cox regression models were further constructed: Model 1 adjusted for age, gender, and ethnicity; Model 2 incorporated additional variables, including education level, frequency of alcohol consumption, smoking status and BMI; Model 3 included all 11 covariates for a comprehensive analysis. In the multivariate Cox regression analysis, we compared the incidence of PD among CD, UC and IBD-U and healthy controls, respectively. In addition, an interaction term between IBD status and PRS was created to examine whether the risk of PD is modified when genetic predisposition are considered. Further stratified analyses were performed by age and categorical variable to explore how the relationship between IBD status and PD risk might vary across subgroups. Finally, a sensitivity analysis was performed using PSM (nearest neighbor ratio of 1:1 without replacement and a caliper width of 0.02) to ensure robustness of the findings after adjusting for confounding factors. In addition, prodromal symptoms of PD, such as REM sleep behavior disorder, olfaction dysfunction, and autonomic nerve impairment, often appear years before a formal clinical diagnosis, so it is possible that some PD patients were prodromal at baseline and were assigned to be healthy controls (Berg et al., 2021). Then after excluding participants who developed PD within 5 to 10 years following the baseline, we re-conducted our analysis using multivariable Cox regression models. The Schoenfeld residual test was used to validate the proportional hazards assumption, and all statistical analyses were executed in R version 4.3.0, with *p* < 0.05 considered significant.

## 3 Results

### 3.1 Baseline characteristics

In the present study, 425,112 participants in total were included, comprising 421,825 individuals in the healthy control group and 3,287 patients in the IBD group (Supplementary Table 2). The latter group included 956 individuals with CD, 1,980 with UC, and 351 with IBD-U, whereas 29 participants from the IBD cohort were diagnosed with PD during the follow-up period. Over an average follow-up period of 13.92 years, there were 2,821 newly diagnosed cases of PD; however, no statistically significant differences were observed between the two groups in terms of gender, Townsend Deprivation Index or PRS. The proportion of White individuals (96.8% vs. 94.8%) and those with a non-university education level (70.3% vs. 66.5%) was higher in the IBD group compared to the healthy control group, which exhibited a larger proportion of individuals who never smoked (55.4% vs. 47.8%) and had a higher frequency of alcohol consumption (20.7% vs. 18.4%) compared to the IBD group. Moreover, within the healthy control group, a greater percentage of participants were without diabetes (91.8% vs. 88.9%) and hypertension (70.3% vs. 65.6%).

### 3.2 Association between IBD and PD

**Figure 2:**
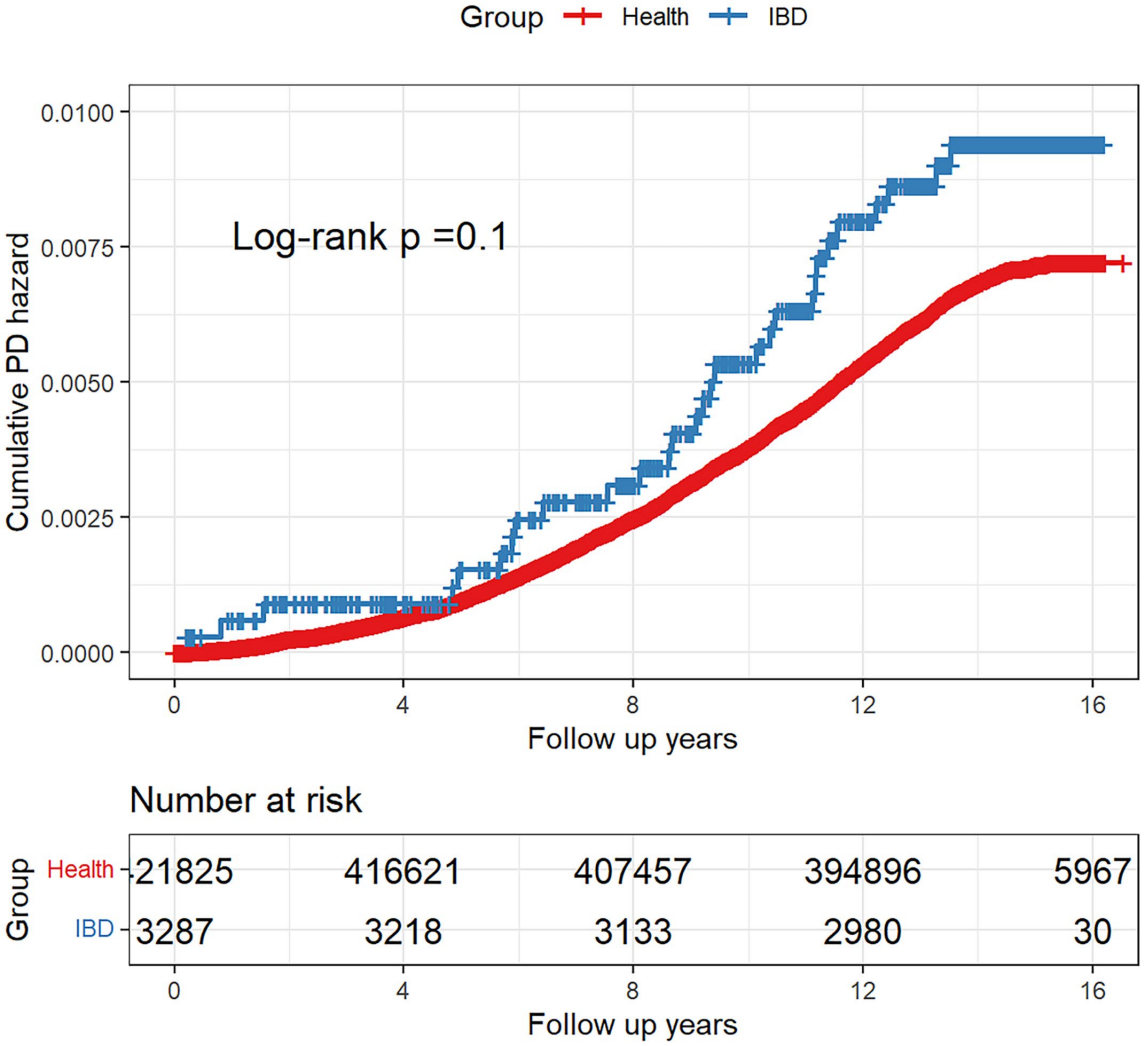
Kaplan–Meier plot for the cumulative probability of PD risk.

**Figure 3:**
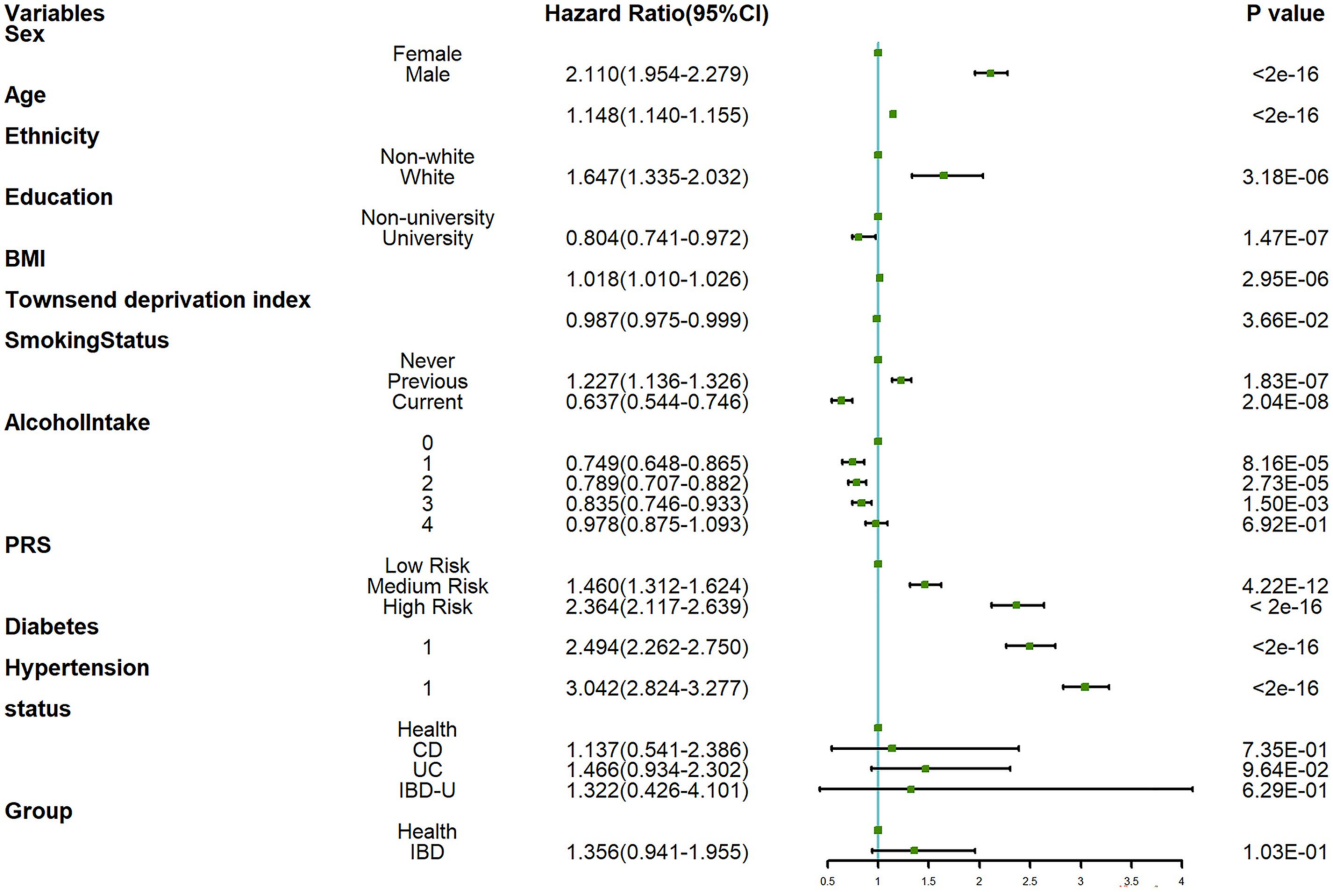
Forest plot for univariate Cox regression analysis.

Based on the Kaplan–Meier method, during an average follow-up period of 13.93 years, there was no significant difference in the risk of developing PD between the healthy control group and the IBD group (Log-rank: *p* = 0.1) (Figure 2). Further, the Schoenfeld residual test indicated that the proportional hazards assumption was met in both groups. According to the results of the univariate Cox regression analysis (Figure 3), the IBD group exhibited an elevated but statistically non-significant risk of developing PD compared to the healthy control group (HR: 1.356, 95% CI: 0.941–1.955, *p* = 0.103), whereas in the three IBD subgroups (CD, UC, and IBD-U), we independently investigated their respective associations with PD employing multivariable Cox regression models for each subgroup, and consistent conclusions were obtained across all three categories.

**Figure 4:**
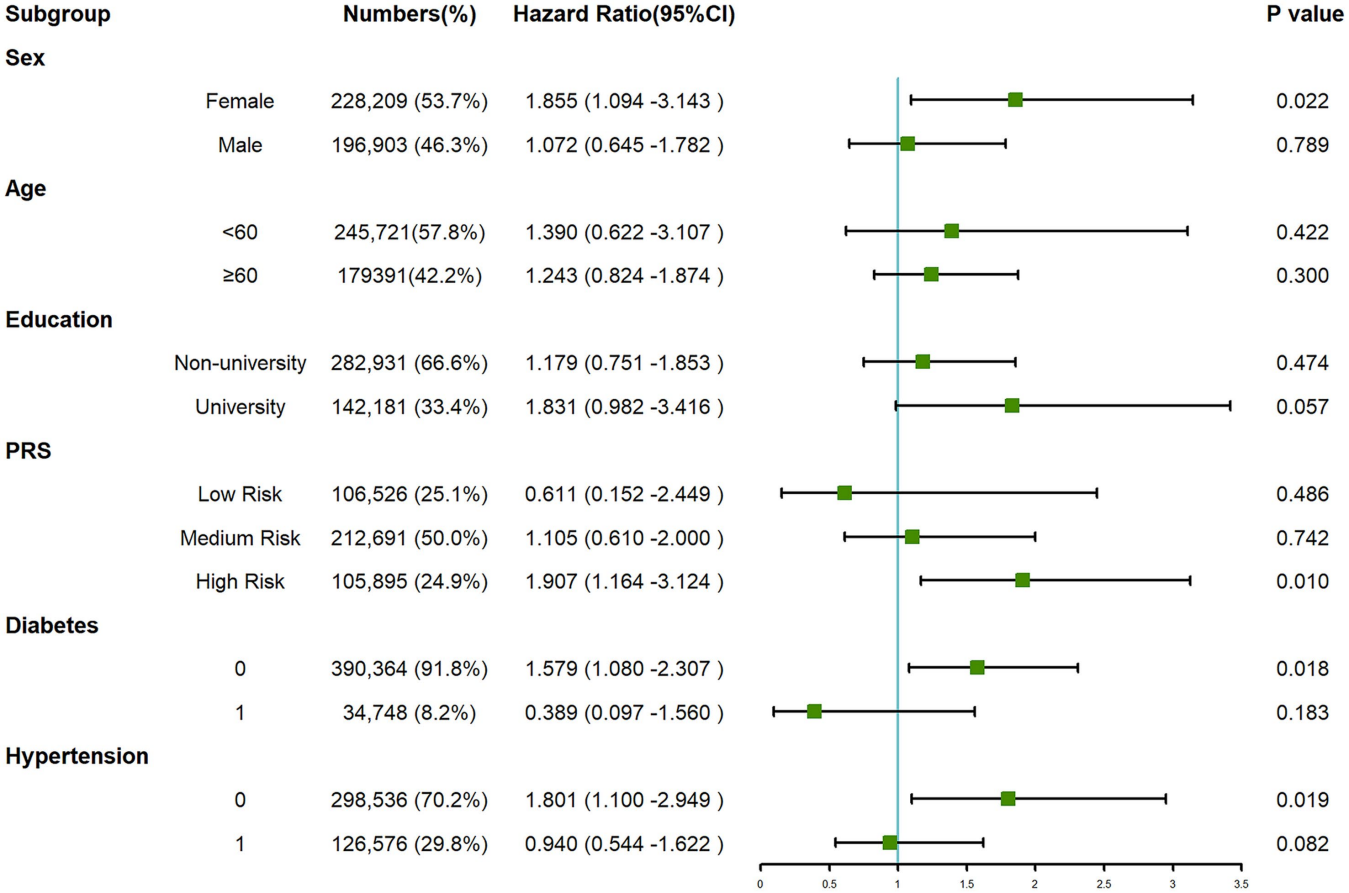
Forest plot for subgroup analysis between IBD and PD.

**Figure 5:**
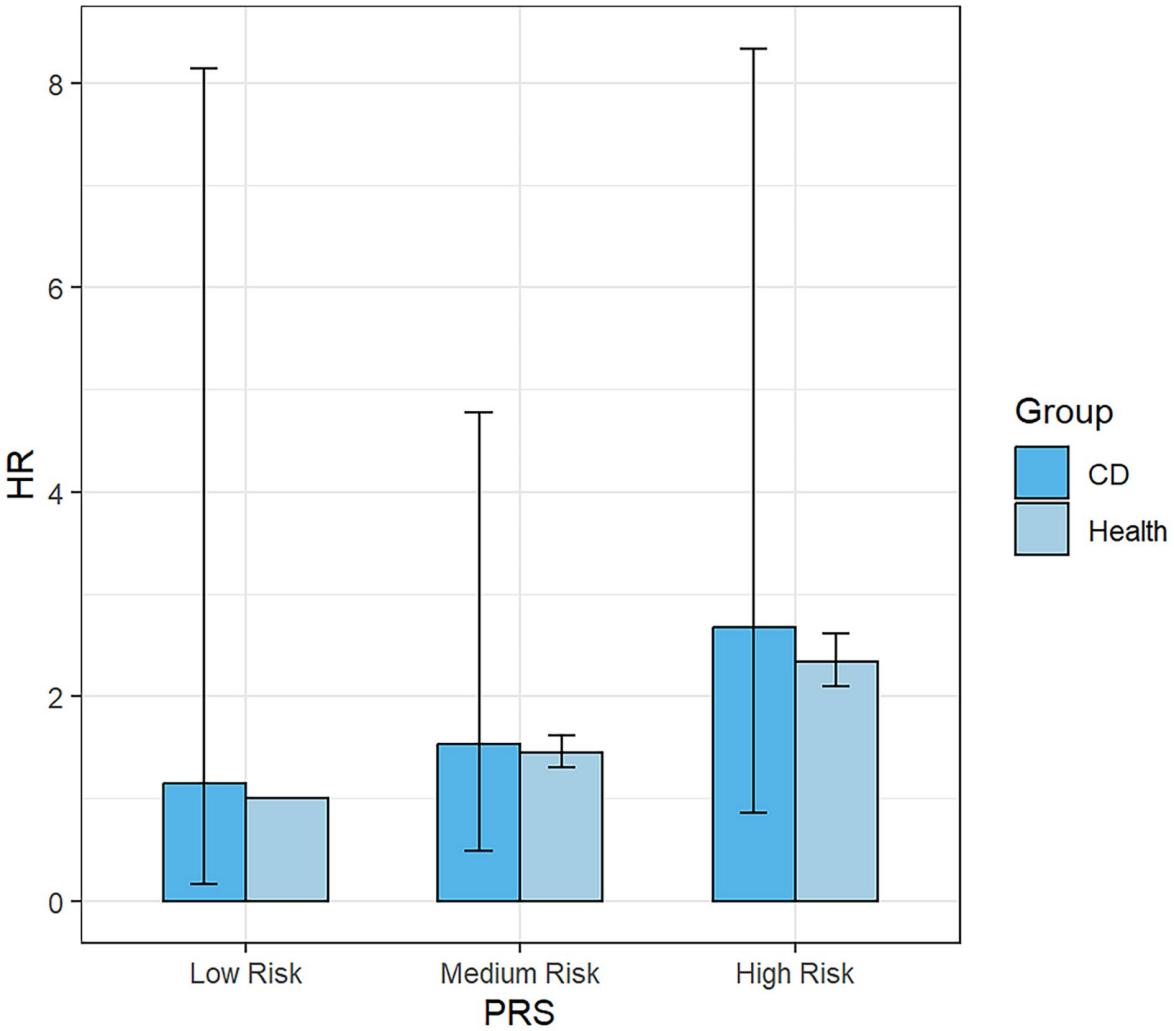
Bar Chart for the impact of the interaction term created by UC status and PRS on the risk of PD. UC, ulcerative colitis; PRS, polygenic risk score.

**Figure 6:**
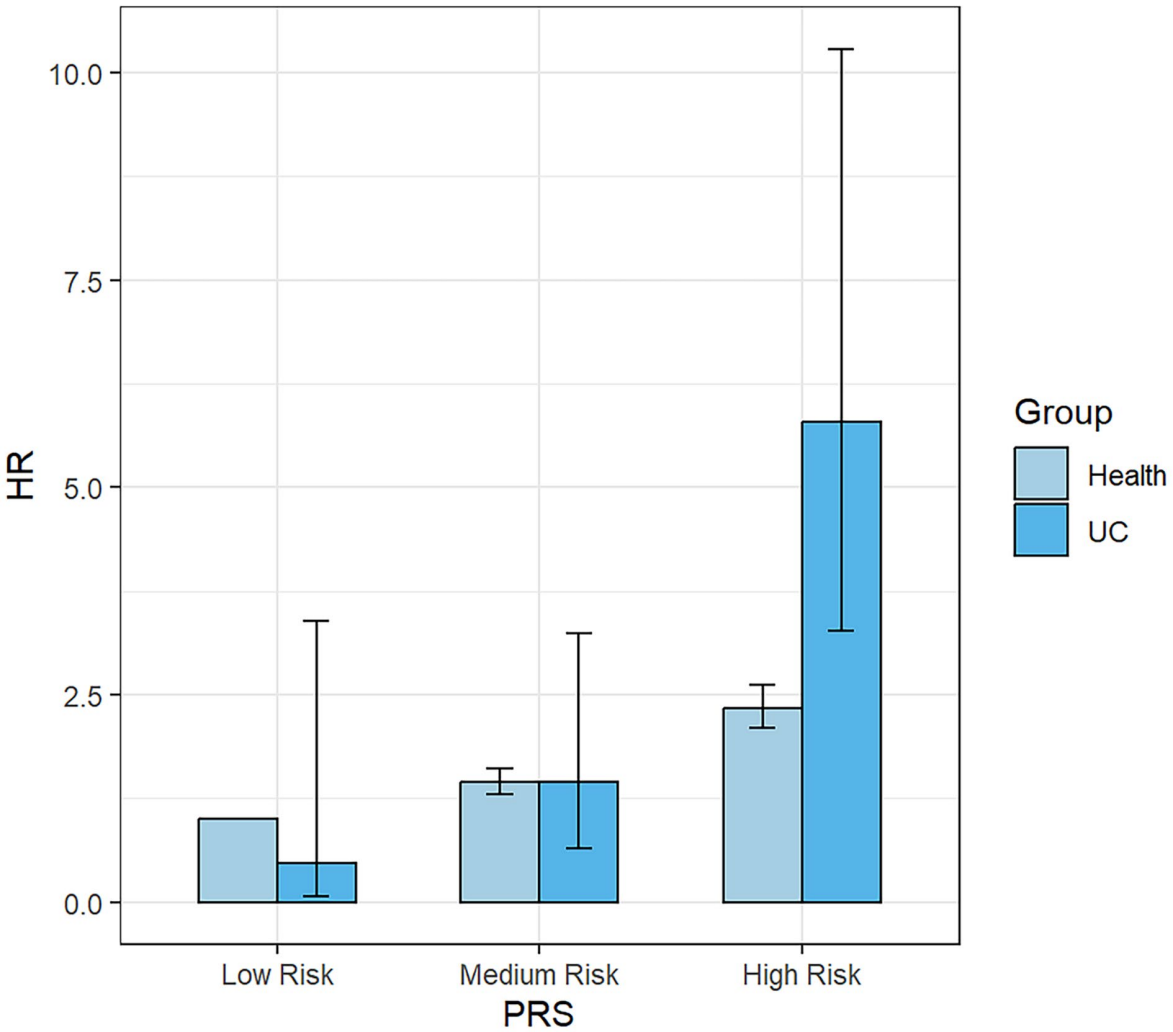
Bar Chart for the impact of the interaction term created by CD status and PRS on the risk of PD. CD, Crohn’s disease.

The subgroup analysis (Figure 4) revealed that within the overall IBD population, the risk of developing PD was elevated among females, those categorized at a high risk of PRS and participants without a history of hypertension and diabetes. The presence of IBD amplified the risk of PD in these specific subgroups. Consequently, we further examined the risk of developing PD within the UC (Supplementary Figure 1) and CD (Supplementary Figure 2) populations. Our findings suggest that the occurrence of PD was not influenced by the presence of CD. However, among UC patients, we still observed an increased risk of PD among females, those categorized as “High Risk” in the PRS, and individuals without hypertension. Nonetheless, the interaction term created to examine the effects of having CD (Figure 5) or UC (Figure 6) along with the genetic risk did not yield significant results for interactive effects.

The sensitivity analysis was conducted utilizing PSM to attenuate the impact of confounding variables. The 1:1 matching was successfully executed for all 3,287 participants (Supplementary Table 3). Aside from drinking frequency and BMI, no statistically significant difference was observed in the baseline characteristics between the matched healthy control and IBD groups. In addition, a subsequent survival analysis revealed that even among the matched cohorts, there remained no statistically significant divergence in the risk of developing PD between the two groups (Supplementary Figure 3). In addition, upon excluding patients who were diagnosed with PD within 5 (*n* = 411) or 10 years (*n* = 1,576) of the baseline, our analysis also did revealed no connection between IBD and PD (Supplementary Tables 4, 5), which might suggest that even when considering a longer period prior to diagnosis, there is no association between IBD and PD.

## 4 Discussion

Throughout disease progression, the motor symptoms (tremor, myotonia, motor retardation and postural balance disorders, etc.) and non-motor symptoms (sleep disorders, autonomic nervous dysfunction, mental and behavioral abnormalities, etc.) of PD will gradually increase, seriously affecting the quality of life of PD patients (Santos Garcia et al., 2021). Meanwhile, IBD encompasses two primary subtypes: CD and UC (Miyoshi and Chang, 2017), where the latter restricted to the colon, characterized by superficial mucosal inflammation that extends continuously proximally, potentially resulting in ulcers, significant bleeding, and toxic megacolon. In contrast, CD can affect any part of the digestive tract and is typically marked by discontinuous lesions. Its hallmark is transmural inflammation, which can lead to complications such as fibrosis, stenosis, fistulae and abscesses (Khor et al., 2011; Wright et al., 2018). Pathophysiological changes in the gastrointestinal system have also been implicated in the onset of PD (Böttner et al., 2012; Fasano et al., 2015), where, increasingly, empirical evidence supports the hypothesis that PD may have its etiological origins in the gastrointestinal tract (Sampson et al., 2016; Knudsen et al., 2018). Beyond the potential common risk gene LRRK2, a genome-wide association study (GWAS) identified an overlap of seven genes presenting a risk for PD with CD (MROH3P, HLA, CCNY, LRRK2, APT, SYMPK and RSPH6A) and four with UC (GUCY1A3, HLA, BTNL2 and TRIM10, Witoelar et al., 2017). Biological pathways involving gut microbiota, autoimmunity, mitochondrial functionality, autophagy and lysosomal activity may forge a close connection between IBD and PD (Lee et al., 2021), but despite the inconclusive role of IBD in predisposing individuals to PD in the present study cohort, the association between these two disorders remains an open question warranting further investigation.

During the 13.93-year follow-up period in this study, 2,821 new cases of PD in total were diagnosed. In response, a univariate Cox regression analysis indicated no significant association between IBD and PD (HR:1.356, 95% CI: 0.941–1.955, *p* = 0.103). When further dissecting the IBD group into CD, UC, and IBD-U, no significant relationships with PD were observed, findings that are consistent even after adjusting for confounding variables across three distinct multivariate models. IBD and PD also show marked differences in clinical presentations among different genders (Cerri et al., 2019). Nonetheless, in subgroup analyses, distinct populations with IBD showed an elevated risk of PD, especially females (HR: 1.855, 95% CI: 1.094–3.143, *p* = 0.022). Based on previous research, UC is more prevalent in male patients (Goodman et al., 2020), while in the case of PD the incidence rate in males is more than double that in females. The reasons for the observed increase in PD risk among females remain unclear, but similarly, in two analogous studies that performed subgroup analyses, an elevated incidence of PD was noted among female with IBD (Park et al., 2019; Villumsen et al., 2019). Existing studies present an inconclusive correlation between hypertension and PD risk (Paganini-Hill, 2001; Ng et al., 2021), the use of antihypertensive agents, specifically calcium channel blockers (Becker et al., 2008), has been implicated in reducing the incidence of PD. Meanwhile, diabetes might elevate the risk of PD (Hu et al., 2007; Pagano et al., 2018), but this association appears to be potentially linked to BMI (Foltynie and Athauda, 2020). In our research, those without hypertension (HR: 1.801, 95% CI: 1.100–2.949, *p* = 0.019) or diabetes (HR: 1.579, 95% CI: 1.080–2.307, *p* = 0.018) also face an increased risk, possibly indicating that unique inflammatory or immune pathways in IBD contribute to neurodegenerative changes. The specific mechanisms remain unclear, warranting further investigation, but nterestingly, this elevated risk was not observed in the CD subgroup, despite remaining significant in the UC subgroup.

Our findings are consistent with several previous studies. For instance, existing Mendelian randomization studies do not support a causal relationship between UC, CD, and PD (Freuer and Meisinger, 2022; Li and Wen, 2022; Zeng et al., 2023). Further, one cross-sectional study found no difference in the co-occurrence of PD between IBD patients and non-IBD controls (Bahler et al., 2017), whereas a nationwide epidemiological survey from Sweden examined the risk of developing PD subsequent to autoimmune diseases and found that neither CD nor UC increased the incidence of subsequent PD diagnosis (Li et al., 2012). Another nationwide cohort study in Sweden from 2002 to 2004, which included 39,652 IBD participants, suggested that UC increases the risk of PD (HR: 1.3, 95% CI: 1.0–1.7, *p* = 0.04), but this association disappeared upon adjusting for the number of medical visits as a covariate (Weimers et al., 2019). Further, one retrospective cohort study using the Truven Health Markets can database included 154,051 IBD patients and an equal number of controls. After adjusting for age, gender, residence type, US region, comorbidities, and behavior, they found no statistically significant association between the two diseases (Coates et al., 2022). However, several studies have reached conflicting conclusions. In another retrospective cohort study, 144,018 patients with IBD were matched with a healthy control group at a 1: 5 ratio, based on age, gender, and the index year of IBD diagnosis and the findings suggest that IBD patients had a higher incidence rate of PD compared to matched controls (RR: 1.28, 95% CI: 1.14–1.44, *p* < 0.001) (Peter et al., 2018). In addition, in a study conducted on the Korean population, which included 24,830 patients with IBD and 99,320 non-IBD controls (Kim et al., 2022), the analysis revealed an increased risk of PD among IBD patients. Meanwhile, in two independent national cohort studies from Danish and Taiwan (Lin et al., 2016; Villumsen et al., 2019), after adjusting for key confounding factors such as age, gender and comorbid conditions, the results from both studies converged to indicate an increased risk of PD among patients with IBD. In another study that included 38,861 individuals with IBD and a control group —matched at a 1:3 ratio for the age and gender—consistent results were obtained. Further, a case–control study and another Spanish cross-sectional study, which utilized 5-aminosalicylic acid (5-ASA) as a proxy for possible IBD groups, proposed that IBD might exert a protective effect against PD (Camacho-Soto et al., 2018; Pinel Rios et al., 2019). Finally, we summarized the previous studies into a table (Supplementary Table 6). Given the inconsistencies in previous studies, our research contributes to the existing body of knowledge. Thus, future endeavors are essential to delve deeper into the specific mechanisms involved.

Compared to previous studies, our research utilized participant data from the UK Biobank and employed Cox Proportional Hazards Model to explore the association between IBD and PD. In addition, we carried out case–control matching and adopted an extended follow-up time. At the time of inclusion, we excluded patients with related gastrointestinal diseases to minimize confounding factors, such as irritable bowel syndrome, colorectal tumors and steatorrhea, whereas in the Cox regression analysis, we adjusted for multiple confounding factors such as age, gender, BMI, and the Townsend Deprivation Index. Furthermore, we separately investigated the relationships of UC and CD with PD. Subgroup analyses were employed to examine disparities in disease risk among different populations, and PSM was used to control for confounding factors. However, while our study adds to the body of literature examining the relationship between IBD and PD, certain limitations should be considered when interpreting our findings and designing future research. First, the sample size of IBD patients was relatively small, consisting of only 3,287 participants. Second, some baseline characteristics of the participants may have changed during the follow-up period, such as BMI, smoking status, and alcohol consumption, which could introduce bias. In addition, both IBD and PD were identified based on ICD-10 coding, which may introduce potential coding biases.

In summary, we confirm no significant association between IBD and PD, except in females (HR:1.989, 95% CI: 1.032–3.835, *p* = 0.040), PRS is at high risk (HR:2.476, 95% CI: 1.401–4.374, *p* = 0.002), participants without hypertension (HR:2.042, 95% CI: 1.128–3.697, *p* = 0.018), C was associated with an increased risk of PD.

## Data availability statement

The data analyzed in this study is subject to the following licenses/restrictions: Researchers may obtain access to the UK Biobank dataset upon application. Requests to access these datasets should be directed to https://biobank.ndph.ox.ac.uk/.

## Author contributions

H-lW: Writing – original draft, Writing – review & editing, Formal analysis. Z-yW: Writing – original draft, Methodology. JT: Writing – original draft, Visualization. D-rM: Writing – original draft, Data curation. C-hS: Writing – original draft, Funding acquisition, Supervision.

## Footnotes

^1^https://biobank.ndph.ox.ac.uk/
