# Supplementary material for: Association between inflammatory bowel disease and Parkinson’s disease: a prospective cohort study of 468,556 UK biobank participants
Source: Front Aging Neurosci. 2024 Jan 15;15:1294879. doi: 10.3389/fnagi.2023.1294879 (PMC10822879; doi:10.3389/fnagi.2023.1294879)
Supplement: Supplementary file 1 [file Data_Sheet_1.docx]

***Supplementary Material***

**Association Between** **Inflammatory Bowel Disease and** **Parkinson’s disease: A Prospective Cohort Study of 468,556 UK Biobank Participants**

1. **Supplementary Figures and Tables**
   1. **Supplementary Figures**


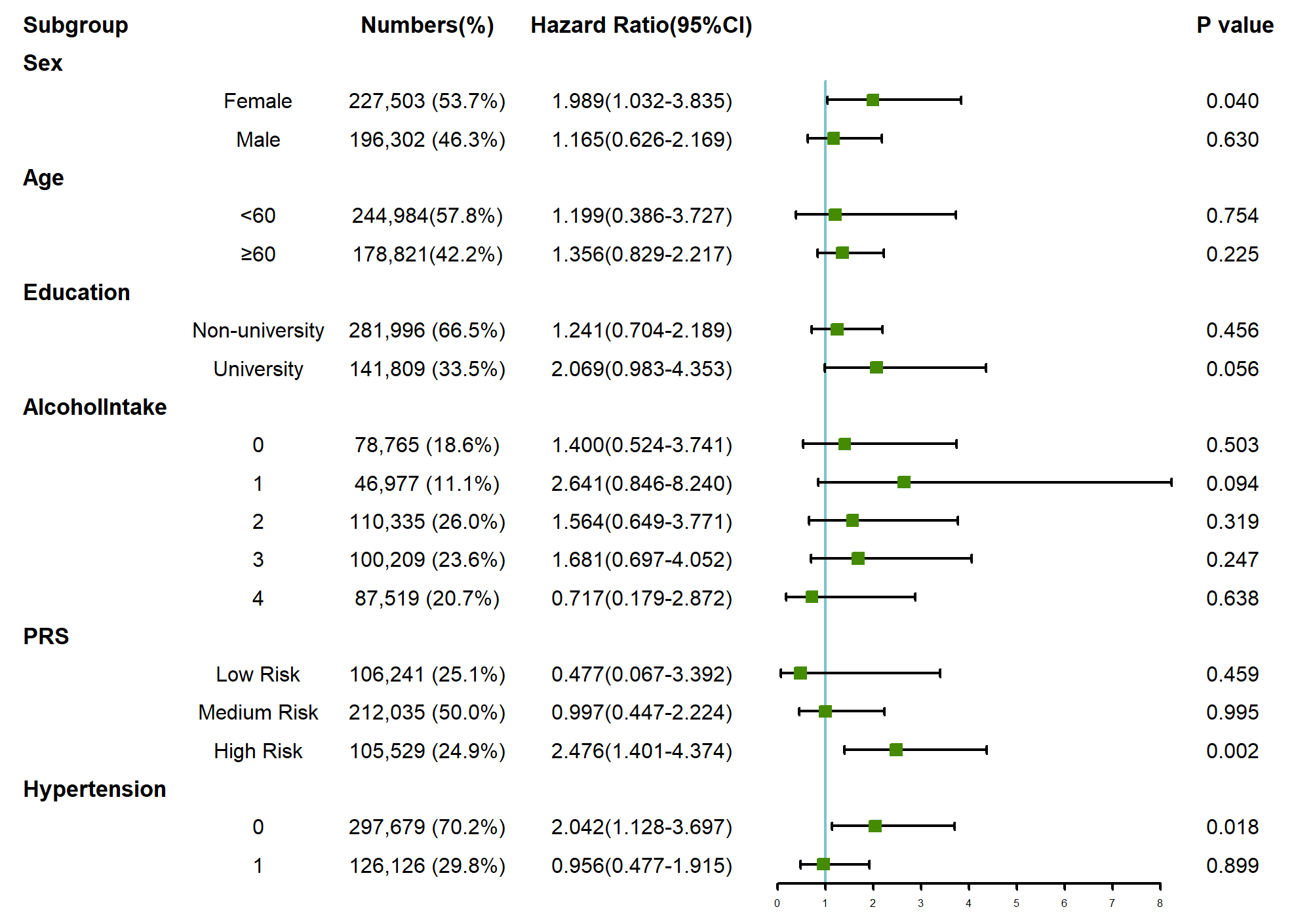


**Supplementary Figure 1.** Forest plot for subgroup analysis between UC and PD.


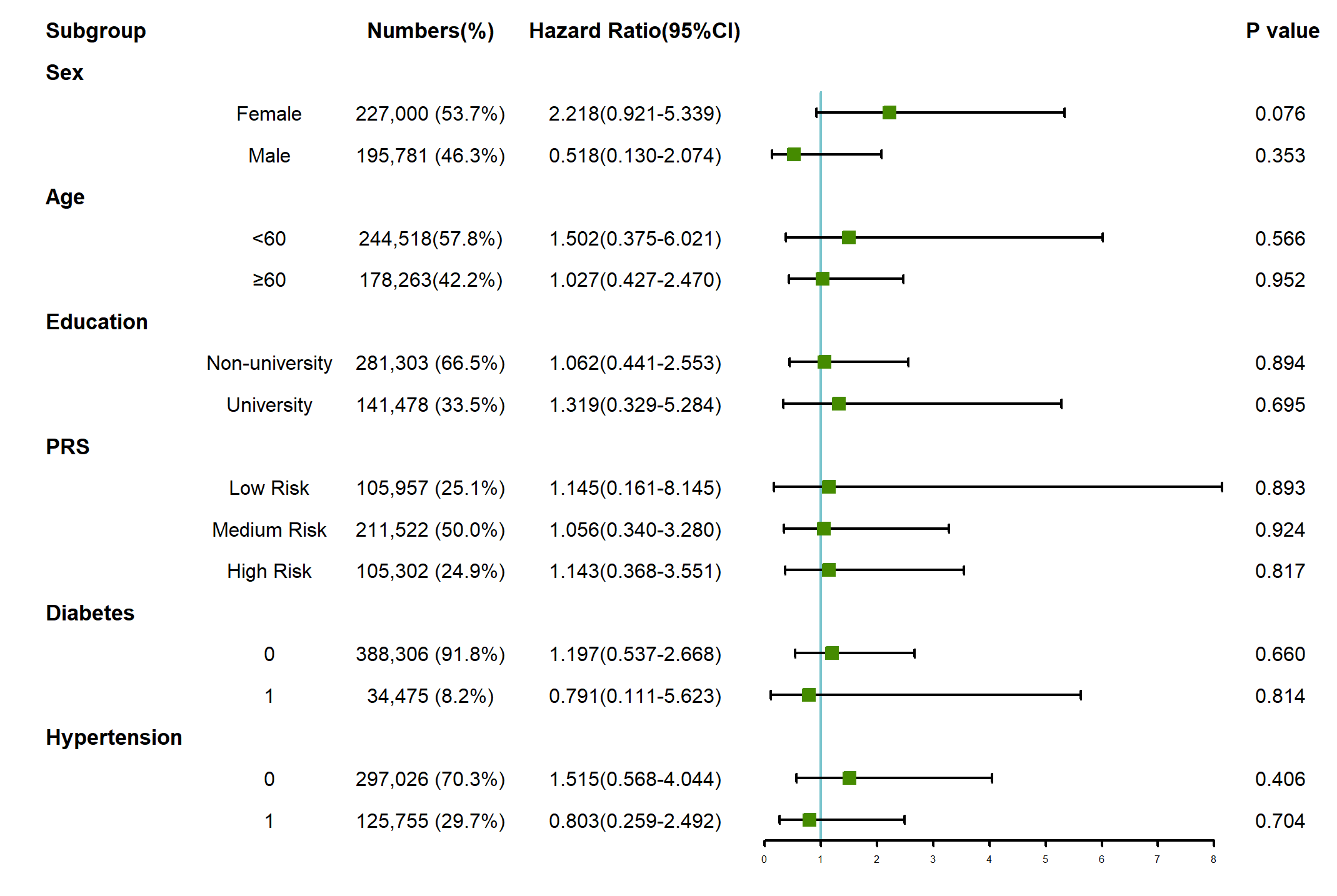


**Supplementary Figure 2.** Forest plot for subgroup analysis between CD and PD.


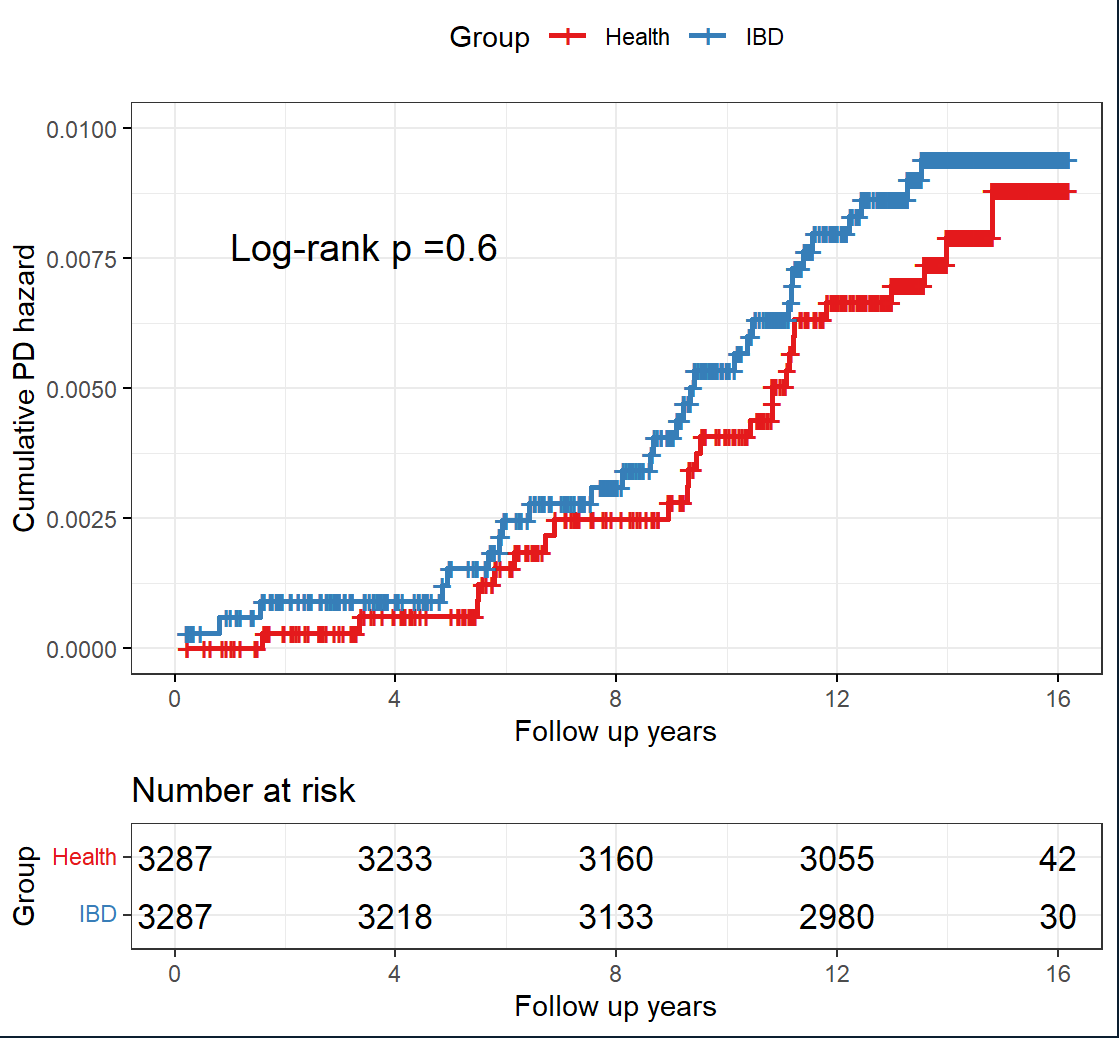


**Supplementary Figure 3.** Kaplan-Meier plot for the cumulative probability of PD risk after PSM.PSM, propensity matching score.

- 1. **Supplementary Tables**

**Supplementary Table 1.** ICD-10 codes used for identification of exclusive diagnoses.

| Exclusive diagnoses |  |
| --- | --- |
| Irritable bowel syndrome | K58 |
| Other noninfective gastroenteritis and colitis | K52 |
| Celiac disease | K90.0 |
| Colorectal neoplasms | C18, C19, C20, C21, C78.5, D01 |

**Supplementary Table 2.** Baseline characteristics of the final studypopulation and incidence of Parkinson’s disease. Continuous variables are shown as means and standard deviations, categorical variables are shown as percentages.

| Variables | Levels | Health  (N=421,825) | IBD (N=3287) | | | P Value |
| --- | --- | --- | --- | --- | --- | --- |
|  |  |  | CD  (N=956) | UC  (N=1980) | IBD-U  (N=351) |  |
| Gender | Female | 226,473 (53.7%) | 527 (55.1%) | 1,030 (52%) | 179 (51%) | .325 |
|  | Male | 195,352 (46.3%) | 429 (44.9%) | 950 (48%) | 172 (49%) |  |
| Age | Mean ± SD | 56.32 ± 8.10 | 56.36 ± 8.26 | 57.51 ± 7.77 | 56.91 ± 7.80 | **<.001** |
| Ethnicity | Non-white | 21953 (5.2%) | 26 (2.7%) | 63 (3.2%) | 16 (4.6%) | **<.001** |
|  | White | 399,872 (94.8%) | 930 (97.3%) | 1,917 (96.8%) | 335 (95.4%) |  |
| Education | Non-university | 280,619 (66.5%) | 684 (71.5%) | 1,377 (69.5%) | 251 (71.5%) | **<.001** |
|  | University | 141,206 (33.5%) | 272 (28.5%) | 603 (30.5%) | 100 (28.5%) |  |
| BMI | Mean ± SD | 27.36 ± 4.73 | 26.64 ± 4.58 | 27.09 ± 4.54 | 27.26 ± 4.94 | **<.001** |
| Townsend deprivation index | Mean ± SD | -1.37 ± 3.06 | -1.33 ± 3.08 | -1.51 ± 2.98 | -1.12 ± 3.12 | .380 |
| Smoking Status | Never | 233,528 (55.4%) | 429 (44.9%) | 990 (50%) | 153 (43.6%) | **<.001** |
|  | Previous | 144,617 (34.3%) | 390 (40.8%) | 860 (43.4%) | 160 (45.6%) |  |
|  | Current | 43,680 (10.4%) | 137 (14.3%) | 130 (6.6%) | 38 (10.8%) |  |
| Alcohol Intake | 0 | 78,376 (18.6%) | 236 (24.7%) | 389 (19.6%) | 70 (19.9%) | **<.001** |
|  | 1 | 46,773 (11.1%) | 128 (13.4%) | 204 (10.3%) | 46 (13.1%) |  |
|  | 2 | 109,801 (26%) | 241 (25.2%) | 534 (27%) | 86 (24.5%) |  |
|  | 3 | 99,741 (23.6%) | 197 (20.6%) | 468 (23.6%) | 83 (23.6%) |  |
|  | 4 | 87,134 (20.7%) | 154 (16.1%) | 385 (19.4%) | 66 (18.8%) |  |
| PRS | Low Risk | 105,747 (25.1%) | 210 (22%) | 494 (24.9%) | 75 (21.4%) | .084 |
|  | Medium Risk | 211,047 (50%) | 475 (49.7%) | 988 (49.9%) | 181 (51.6%) |  |
|  | High Risk | 105,031 (24.9%) | 271 (28.3%) | 498 (25.2%) | 95 (27.1%) |  |
| Diabetes | 0 | 387,441 (91.8%) | 865 (90.5%) | 1,759 (88.8%) | 299 (85.2%) | **<.001** |
|  | 1 | 34,384 (8.2%) | 91 (9.5%) | 221 (11.2%) | 52 (14.8%) |  |
| Hypertension | 0 | 296,379 (70.3%) | 647 (67.7%) | 1,300 (65.7%) | 210 (59.8%) | **<.001** |
|  | 1 | 125,446 (29.7%) | 309 (32.3%) | 680 (34.3%) | 141 (40.2%) |  |
| PD | 0 | 419,033 (99.3%) | 949 (99.3%) | 1,961 (99%) | 348 (99.1%) | .149 |
|  | 1 | 2,792 (0.7%) | 7 (0.7%) | 19 (1%) | 3 (0.9%) |  |
| time | Mean ± SD | 13.93 ± 2.09 | 13.66 ± 2.66 | 13.81 ± 2.34 | 13.70 ± 2.39 | <.001 |

**Supplementary Table 3.** Baseline characteristics of the final studypopulation and incidence of Parkinson’s disease after PSM.

| Variables | Levels | Health (N=3287) | IBD (N=3287) | p |
| --- | --- | --- | --- | --- |
| Gender | Female | 1,736 (52.8%) | 1,736 (52.8%) | 1.000 |
|  | Male | 1,551 (47.2%) | 1,551 (47.2%) |  |
| Age | Mean ± SD | 57.11 ± 7.93 | 57.11 ± 7.93 | .991 |
| Ethnicity | Non-white | 103 (3.1%) | 105 (3.2%) | .944 |
|  | White | 3,184 (96.9%) | 3,182 (96.8%) |  |
| Education | Non-university | 2,316 (70.5%) | 2,312 (70.3%) | .935 |
|  | University | 971 (29.5%) | 975 (29.7%) |  |
| BMI | Mean ± SD | 27.70 ± 4.92 | 26.98 ± 4.60 | <.001 |
| Townsend deprivation index | Mean ± SD | -1.35 ± 3.10 | -1.41 ± 3.03 | .443 |
| Smoking Status | Never | 1,575 (47.9%) | 1,572 (47.8%) | .997 |
|  | Previous | 1,408 (42.8%) | 1,410 (42.9%) |  |
|  | Current | 304 (9.2%) | 305 (9.3%) |  |
| Alcohol Intake | 0 | 588 (17.9%) | 695 (21.1%) | .001 |
|  | 1 | 344 (10.5%) | 378 (11.5%) |  |
|  | 2 | 913 (27.8%) | 861 (26.2%) |  |
|  | 3 | 749 (22.8%) | 748 (22.8%) |  |
|  | 4 | 693 (21.1%) | 605 (18.4%) |  |
| PRS | Low Risk | 779 (23.7%) | 779 (23.7%) | 1.000 |
|  | Medium Risk | 1,644 (50%) | 1,644 (50%) |  |
|  | High Risk | 864 (26.3%) | 864 (26.3%) |  |
| Diabetes | 0 | 2,927 (89%) | 2,923 (88.9%) | .906 |
|  | 1 | 360 (11%) | 364 (11.1%) |  |
| Hypertension | 0 | 2,154 (65.5%) | 2,157 (65.6%) | .959 |
|  | 1 | 1,133 (34.5%) | 1,130 (34.4%) |  |
| PD | 0 | 3,262 (99.2%) | 3,258 (99.1%) | .682 |
|  | 1 | 25 (0.8%) | 29 (0.9%) |  |
| status | Health | 3,287 (100%) | 0 (0%) | <.001 |
|  | CD | 0 (0%) | 956 (29.1%) |  |
|  | UC | 0 (0%) | 1,980 (60.2%) |  |
|  | IBD-U | 0 (0%) | 351 (10.7%) |  |
| time | Mean ± SD | 13.86 ± 2.23 | 13.76 ± 2.44 | .068 |

**Supplementary Table 4. Multivariate analysis for incidence of PD in participants with CD,UC and IBD-U after excluding participants with PD within 5 years after baseline**

Model 1 was adjusted for age, gender, and ethnicity; Model 2 further included education level, alcohol intake, smoking status, and BMI; Model 3 included all 11 covariables for comprehensive analysis.

|  | Model 1 | |  | Model 2 | |  | Model 3 | |
| --- | --- | --- | --- | --- | --- | --- | --- | --- |
| Status | HR with 95%CI | P value |  | HR with 95%CI | P value |  | HR with 95%CI | P value |
| CD | 1.155(0.518-2.574) | 0.724 |  | 1.159(0.520-2.584) | 0.717 |  | 1.097(0.492-2.446) | 0.820 |
| UC | 1.213(0.730-2.016) | 0.456 |  | 1.208(0.727-2.007) | 0.466 |  | 1.165(0.701-1.935) | 0.556 |
| IBD-U | 1.472(0.474-4.566) | 0.504 |  | 1.481(0.477-4.596) | 0.497 |  | 1.331(0.429-4.131) | 0.621 |

**Supplementary Table 5. Multivariate analysis for incidence of PD in participants with CD,UC and IBD-U after excluding participants withPD within 10 years after baseline**

Model 1 was adjusted for age, gender, and ethnicity; Model 2 further included education level, alcohol intake, smoking status, and BMI; Model 3 included all 11 covariables for comprehensive analysis.

|  | Model 1 | |  | Model 2 | |  | Model 3 | |
| --- | --- | --- | --- | --- | --- | --- | --- | --- |
| Status | HR with 95%CI | P value |  | HR with 95%CI | P value |  | HR with 95%CI | P value |
| CD | 1.507(0.565-4.023) | 0.413 |  | 1.520(0.569-4.057) | 0.404 |  | 1.431(0.536-3.820) | 0.474 |
| UC | 1.103(0525-2.319) | 0.796 |  | 1.100(0.523-2.313) | 0.801 |  | 1.053(0.501-1.214) | 0.892 |
| IBD-U | 0.975(0.137-6.925) | 0.980 |  | 0.983(0.138-6.982) | 0.986 |  | 0.881(0.124-6.259) | 0.899 |

**Supplementary Table 6.** Previous Clinical Studies on the Association Between IBD and PD

| Author | Methods | N(All) | N(IBD) | N(PD) | OR/HR for PD (95%CI) |
| --- | --- | --- | --- | --- | --- |
| Bahler C 2017 | Cross-sectional study | 1,119,429 | 4791 | 10,104 | OR:0.92(0.67-1.27) |
| Li X 2012 | Nationwide Epidemiological Study | 310,522 | NA | 932 | Celiac disease (0.13-2.09) |
|  |  |  |  |  | Ulcerative colitis (0.57-1.36) |
| Weimers P 2019 | nationwide cohort study | 436,172 | 39,652 | 1,761 | HR:1.3(1.0-1.7) |
| Coates MD 2022 | retrospective cohort study | 308,102 | 154,051 | 132 | HR:1.01(0.72-1.42) |
| Peter I 2018 | retrospective cohort study | 864,108 | 144,018 | 1,796 | RR: 1.28(1.14-1.44) |
| Kim GH 2022 | nationwide cohort study | 124,150 | 24,830 | 354 | HR:1.56(1.24-1.97) |
| Villumsen M 2022 | nationwide cohort study | 7,624,736 | 76,477 | 40,119 | HR:1.22(1.09-1.35) |
| Lin JC 2016 | Retrospective Cohort Study | 41,865 | 8,373 | 396 | HR:1.40(1.11-1.77) |
| Camacho-Soto A 2018 | Case-control Study | 207,885 | 4,980 | 89,790 | OR: 0.85(0.80-0.91) |
| Pinel Rios J 2019 | Cross-sectional study | 2,020,868 | 7,485 | 19,966 | OR:0.94(0.72-1.23) |
